# 胸腔镜肺癌根治术后早期并发高危肺栓塞介入溶栓治疗经验

**DOI:** 10.3779/j.issn.1009-3419.2018.10.08

**Published:** 2018-10-20

**Authors:** 胜杰 靖, 建明 周, 启同 陆, 鑫 楚, 伟 何, 杰 蒋, 新 薛, 志勇 刘, 涛 薛

**Affiliations:** 210009 南京，东南大学附属中大医院 Department of Toracic Surgery, ZhongDa Hospital Southeast University, NanJing 210009, China

**Keywords:** 肺肿瘤, 肺叶切除术, 高危肺栓塞, 介入治疗, 溶栓, Lung neoplasms, Lobectomy, Massive pulmonary embolism, Interventional therapy, Thrombolysis

## Abstract

**背景与目的:**

肺栓塞（pulmonary thrombosis embolism, PTE）是肺癌根治术围术期严重并发症之一，其中高危PTE因伴有休克、低血压具有病情进展快、死亡率高的特点。这类患者的治疗目前尚无统一标准，是胸外科医生临床工作中的难点。本文通过总结分析我院2例肺癌术后早期并发高危PTE患者的治疗过程，探讨此类病例的诊疗策略，为广大胸外科医生提供参考。

**方法:**

分析2017年在我院接受胸腔镜肺癌根治手术术后早期并发高危PTE患者两例。1例予静脉联合肺动脉介入溶栓治疗，1例予单纯肺动脉介入溶栓治疗。总结分析两例病人治疗效果及治疗过程中出现的并发症。

**结果:**

2例患者均为女性，年龄分别为66岁、61岁，发生PTE的时间分别为术后48 h、45 h，接受介入溶栓时间分别是发病后70 min、50 min，介入治疗时间分别是120 min、100 min，介入溶栓后引流分别是4, 690 mL、520 mL，溶栓后住院时间分别是21 d、14 d，随访6个月均无明显并发症。

**结论:**

肺癌术后早期发生高危PTE建议尽早行肺动脉内介入溶栓治疗，相对于静脉溶栓治疗，肺动脉内介入溶栓具有给药精准、药物用量容易控制、疗效快、出血风险小等优点。

## Abstract (en)

**Background and objective:**

Pulmonary thrombosis embolism (PTE) is one of the most severe complications of perioperative radical mastectomy. Massive PTE is often accompanied by shock and hypotension which is characterized by rapid progression and high mortality. There is no standard for the treatment of these patients, which is thoracic surgery, and it is a critical issue in the thoracic surgeons. This article summarizes and analyzes the treatment of two patients with high-risk PTE at the early stage of postoperative lung cancer in our hospital. In addition, we discusses the diagnosis and treatment strategies of these cases to provide a reference for the thoracic surgeons.

**Methods:**

We presented two patients with high-risk PTE at the early stage after thoracic surgery for radical surgery in our hospital back in 2017. One case was treated with intravenous venous interventional thrombolysis, and the other was treated with thrombolysis alone. The treatment effect of two patients and the complications during the treatment has been recorded to detail and summarized.

**Results:**

Both patients were female who aged 66 and 61 years old. The time point of pulmonary embolism was 48 h and 45 h after operation, and the time of interventional thrombolysis was 70 minutes and 50 minutes after onset respectively. After 120 minutes and 100 minutes, the drainage after interventional thrombolysis was 4, 690 mL and 520 mL respectively. The hospitalization time after thrombolysis was 21 days and 14 days respectively. There was no obvious complication through a follow-up of 6 months.

**Conclusion:**

Early postoperative acute massive pulmonary embolism in lung cancer should be treated with pulmonary interventional thrombolysis as soon as possible. Compared with intravenous thrombolysis, pulmonary interventional thrombolysis shows accuracy, easy controlling of dosage, fast curative effect and low bleeding risk.

近年来随着胸外科手术技术的发展，肺癌根治术相关的并发症正逐渐减少，但随着辅助检查和对肺栓塞（pulmonary thrombosis embolism, PTE）认识的增加，肺癌术后PTE发病率报道在逐步上升^[1]^。PTE按病情严重程度分为高危PTE和非高危PTE，其中高危PTE以休克和低血压为主要临床表现，具有病情进展快、治疗难度大、死亡率高的特点^[2]^。特别是肺癌术后早期发生的高危PTE死亡率高达44%-93%^[3]^，是PTE治疗的难点。现将我院两例肺癌根治术后早期并发高危PTE患者的救治过程进行分析总结，探讨此类患者的治疗策略。

## 1 资料与方法

### 1.1 病例1

患者女性，66岁。因“咳嗽、咳痰伴低热1周”入院，既往无基础疾病。入院检查诊断：右上肺占位：肺癌？。予排除手术禁忌后行“胸腔镜下右上肺癌根治术”，术后病理证实为右上肺腺癌Ib（T2aN0M0）。术后常规予抗感染、雾化、化痰、双下肢肢体气压治疗等处理。患者于术后28 h开始下床活动，术后48 h上厕所回床时突发晕厥、意识丧失；查体：血压74 mmHg/52 mmHg、心率130次/分，末梢SPO_2_（46%-57%），双侧瞳孔等大等圆，对光反射存在，全身大汗，呼吸急促。立即予以下紧急处理：（1）高流量吸氧，快速补液，应用血管活性药，头部冰袋物理降温；（2）麻醉科会诊气管插管；（3）急查动脉血气示pH 7.295，PCO_2_ 44.2 mmHg，PO_2_ 36.7 mmHg，SpO_2_ 62.3%，乳酸（Lac）4.4 mmol/L D二聚体750 U/L；（4）联系床旁心脏彩超示右心扩大，肺动脉高压，下腔静脉增宽，未探及肺动脉栓子，下肢静脉B超显示肌间静脉曲张，血栓形成；（5）请介入血管外科、呼吸科、重症医学科急会诊。综合考虑患者为急性高危PTE，予低分子肝素（low molecular weight heparin, LMWH）4, 000 IU皮下注射、尿激酶20万IU静脉推注、甲强龙40 mg静脉推注。经过初步治疗患者仍存在低氧血症、低血压，与患者家属沟通后决定即刻行介入治疗。遂于患者发病70 min后行肺动脉介入治疗，造影示双下肺动脉血栓形成（图 1）。立即予旋转碎栓、重组组织型纤溶酶原激活剂（recombinant tissue plasminogen activator, rt-PA）6 mg造影管推入，经介入溶栓处理后患者心率迅速降为120次/分，SPO_2_ 升至90%-95%，意识恢复，再次肺动脉内造影提示双下肺血流较前改善，未见肺动脉活动性出血，继以rt-PA 44 mg在2 h内静脉泵入。后转重症监护病房继续肝素抗凝治疗。溶栓治疗3 h后胸腔引流增多，血小板减少至42×10^9^/L，停用肝素，输注红细胞12.5单位、血浆1, 225 mL及血小板2个治疗量；溶栓治疗48 h后，胸腔出血控制。监测血小板正常，排除肝素介导的血小板减少症，桥接LMWH抗凝，同时口服华法林治疗，血浆凝血酶原时间INR值达到2.3，停用LMWH，单用华法林治疗。10 d后复查CT肺动脉造影（computed tomography pulmonary angiography, CTPA）示右下肺动脉部分充盈缺损，左肺动脉充盈良好（图 2）。病情稳定予以出院，出院后继续口服华法林抗凝治疗6个月，随访无不良并发症。

**1:**
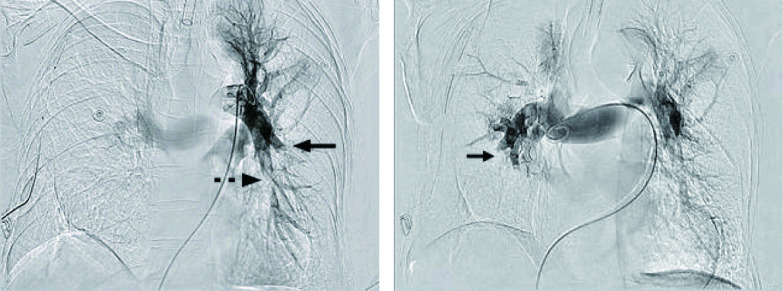
双下肺动脉血栓形成，
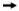
示肺动脉截断，
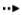
示肺动脉充盈缺损 Double lower pulmonary thrombosis, 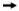 Pulmonary arter y truncation, 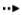 Pulmonary filling defect

**2:**
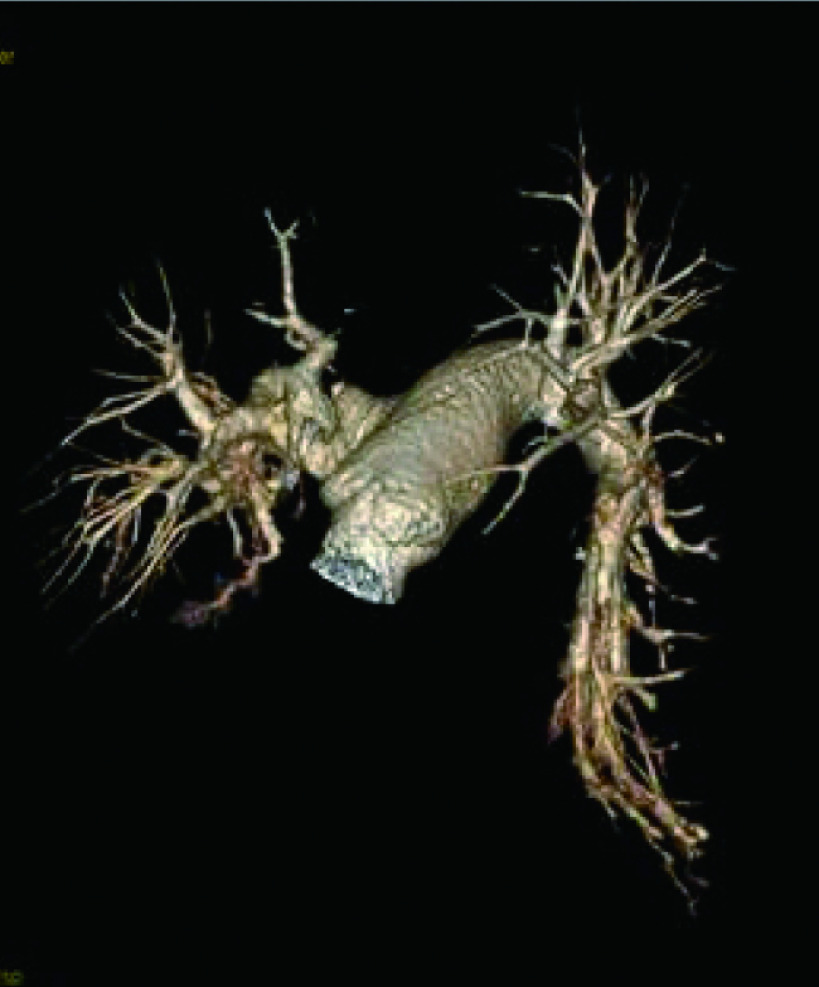
10 d后复查CTPA提示双下肺动脉显影良好 CTPA recommends double pulmonary artery perfusion well

### 1.2 病例2

患者女性，61岁，因“间断上腹痛3个月”首诊收入消化科。既往25年前行直肠癌根治术，高血压病2级病史10年。入院胸部CT提示：右上肺占位：肺癌？转移癌？，遂转胸外科进一步治疗。予排除手术禁忌后行“胸腔镜下右上肺癌根治术”，术后病理：右上肺腺癌Ia（T1aN0M0）。术后予抗感染、雾化、化痰、双下肢肢体气压治疗等处理。术后45 h，患者如厕后突发胸闷气喘、呼吸急促，神志清楚。心电监护脉氧72%、心率105次/分、BP 100 mmHg/50 mmHg。立刻予卧床、高流量吸氧。但患者病情进行性加重，心率逐渐上升至140次/分，血压下降至82 mmHg/48 mmHg，继续予以下处理：（1）储气囊面罩吸氧，LMWH 4, 000 IU皮下注射、氨茶碱0.25静滴、甲强龙40 mg静脉推注，快速补液，应用血管活性药；（2）急查动脉血气PO_2_ 45.6 mmHg及D二聚体3, 395 U/L。（3）请介入血管外科、呼吸科、重症医学科急会诊。考虑患者为急性高危PTE，决定即刻行肺动脉内介入治疗，于患者发病后50 min行肺动脉造影显示左肺动脉栓塞（图 3），予旋转碎栓、rt-PA 10 mg推注，10 min后SPO_2_ 逐渐升至100%，心率100次/分，血压110 mmHg/64 mmHg。再次造影示左肺动脉灌注改善，未见肺血管活动性出血，转入重症监护病房继续予肝素抗凝治疗3 d。病情平稳后转回胸外科病房继续以LMWH抗凝，同时口服华法林治疗，血浆凝血酶原时间INR值达到2.5，停用LMWH，单用华法林治疗。介入溶栓、抗凝治疗未引起手术部位出血。两周后复查CTPA示左上肺动脉少量充盈缺损，余肺动脉通畅（图 4）。病情稳定予以出院，出院后继续口服华法林抗凝治疗6个月，随访无不良并发症。

**3:**
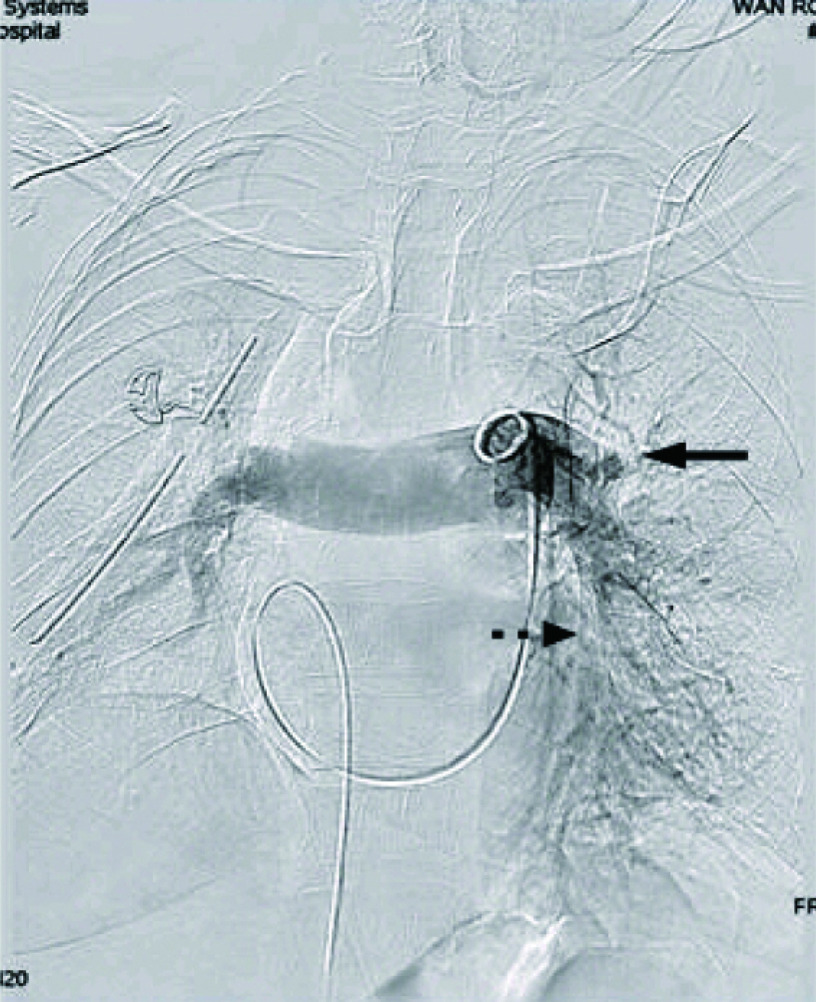
左肺动脉血栓形成，
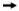
示肺动脉截断，
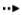
示肺动脉充盈缺损 Left pulmonary thrombosis, 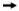 Pulmonary artery truncation, 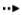 Pulmonary filling defect

**4:**
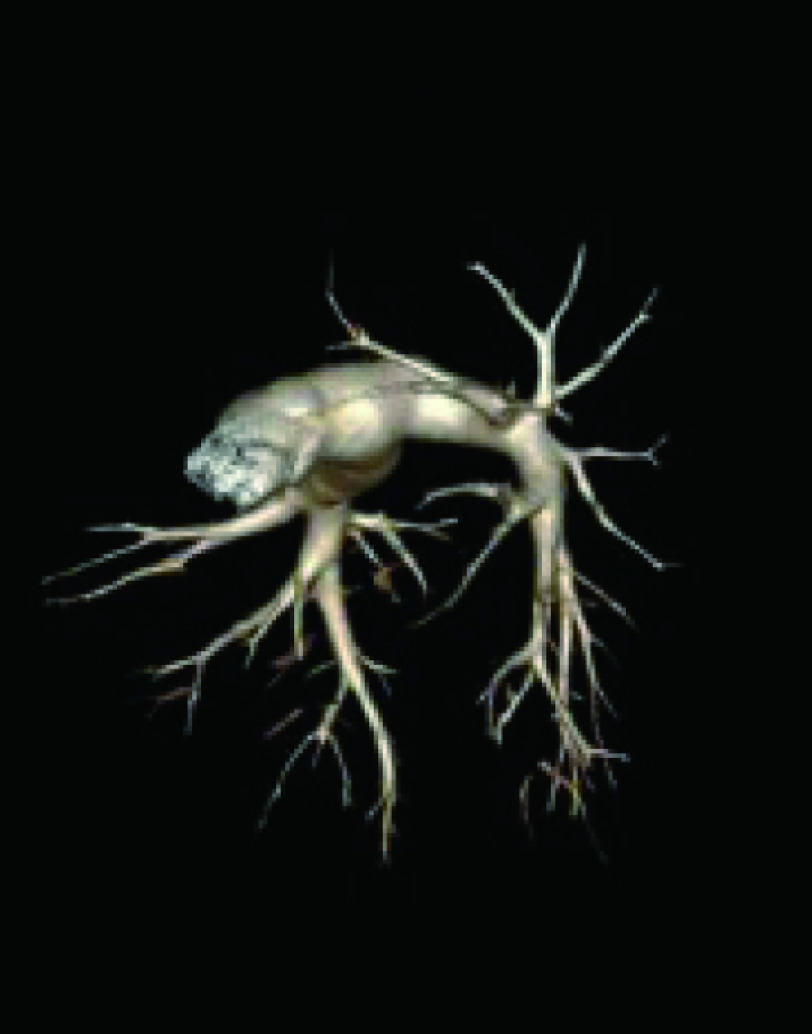
14 d后复查CTPA提示左肺动脉显影良好，仅存左上肺动脉少量充盈缺损 CTPA recommends left pulmonary artery perfusion well, only a small filling defect in the left upper pulmonary artery

## 2 结果

2例患者一般资料详见表 1，发病时特点详见表 2，早期治疗方案详见表 3。其中病例1患者于初始溶栓治疗3 h后出现手术部位出血，予止血治疗，于初始溶栓治疗48 h后，出血控制，总引流量4, 690 mL，于溶栓治疗后第21天出院。病例二采用介入溶栓及抗凝治疗，未出现出血并发症，总引流量520 mL，于介入溶栓治疗后第14天出院。两例患者出院后均继续口服华法林抗凝治疗6个月，随访无不良事件发生。

**1:** 病例特点 Patients'characteristics

Patient	Age	Sex	Operation	Operation time (min)	Bleeding (mL)	Intraoperative transfusion	Pathological diagnosis	Remarks
1	66	F	Lobectomy (RUL) with SMLD	120	150	0	Ad Ib (T2a, N0, M0)	-
2	61	F	Lobectomy (RUL) with SMLD	105	200’	0	Ad Ib (T1a, N0, M0)	Hypertension, rectal cancer
F: female; RUL: right upper lobe; SMLD: systematic mediastinal lymph node dissection; Ad: adenocarcinoma.								

**2:** 发病时病例特点 Characteristics of the acute pulmonary thrombosis embolism onset

Patient	Time of onset (h)(POT)	Trigger of onset	Consciousness	SPO_2_	HR (bpm)	BP (mmHg)	Respiratory rate (/min)	PA
1	48	Defecation	Comatose	46%	130	74/52	Intubation	LLL & RLL
2	45	Defecation	Conscious	72%	140	82/48	40	LL
POT: postoperative time; LLL: left lower lobe; RLL: right lower lobe; LL: left lung.								

**3:** 早期治疗方案 Emergency treatment

Patient	Treatment regimen	Time between onset and Intervention (min)	Time of Intervention (min)	Bleeding (mL)	transfusion
1	IVT: Urokinase 200, 000 U (ⅳ)+ LMWH 4, 100 U (ⅳ)→Intervention: rt-PA 6 mg injection→IVT: rt-PA 44 mg (microinfusion pump 2 h) →Heparin 10, 000 U/h (3 h)	70	120	4, 690	RCS 12.5 u, plasma 1225 mL, platelet 2 therapeutic dose
2	LMWH 4, 100 U (ⅳ) →Intervention: rt-PA 10 mg injection→Heparin10, 000 U/h (3 d)	50	100	520	-
IVT: intravenous thrombolysis; ⅳ: intravenous injection; RCS: red cell suspension.					

## 3 讨论

在全球范围内PTE和深静脉栓塞（deep vein thrombosis, DVT）均有很高的发病率，据我国PTE-DVT防治合作中心1997年-2008年的统计资料显示，PTE在我国平均每年的发病率为1‰^[4]^，来自国内60家大型医院的统计资料显示，住院患者中PTE的比例从1997年的0.26‰上升到2008年的1.45‰^[5]^。PTE也是肺癌手术后常见且危险的并发症之一，Sakuragi等^[6]^持续观察一组行肺癌根治术的肺癌患者，发现这组患者的PTE发病率为19.5%，明显高于其他人群。且这类病人的死亡风险也较高，据报道单肺叶切除术后并发急性PTE的死亡率约为22%，而全肺切除的患者并发急性PTE的死亡率更是高达50%。

肺癌根治术后患者并发PTE更容易表现为高危PTE，因为行肺癌根治术的患者至少缺少一叶肺甚至一侧肺的血管床，累及同样肺血管的PTE对肺癌术后患者的影响更大。对于急性高危PTE的治疗方法，目前指南普遍建议溶栓或肺动脉血栓摘除术联合抗凝治疗。溶栓治疗的主要风险是并发致命性出血，特别是近期接受过重大手术治疗的PTE患者^[7]^。手术取栓可以作为在溶栓治疗失败或溶栓绝对禁忌证患者的最终选择，但同样面临着高死亡率，约43%-84%^[8]^。因此致命性高危PTE，缺少安全有效的治疗办法，溶栓治疗仍是其首选的治疗办法。在病例一的治疗方案中，我们在介入治疗前后均使用静脉溶栓治疗，虽然PTE的治疗效果显著，但患者很快出现了手术部位的活动性出血。有研究^[9]^报道PTE行静脉溶栓治疗后发生出血的概率约20%，虽然没有关于胸部手术术后早期出现PTE行静脉溶栓治疗出血风险的研究，但我们相信这只会将更高。本例病人经过停用肝素、成份输血、纠正低血小板等治疗后出血停止且未出现PTE加重，最终转危为安。因此如何能降低溶栓治疗的出血并发症是这类病人治疗成功的关键，在没有革命性药物出现之前我们能改进的只有用药途径和剂量。在病例二的治疗过程中我们吸取教训，没有静脉应用溶栓药，仅在介入治疗过程中于靶血管内给药，在纠正休克、低血压状态后及时停用溶栓剂，改用肝素抗凝治疗，降低了患者的出血风险。

我们这两例病人抢救成功不仅仅是选择了介入溶栓治疗这一点，更为重要的是能够做到医疗与护理、临床与医技、多学科之间的紧密协作，在诊断、转运、治疗各个环节做到预先安排、无缝对接，最终挽救患者的生命。这得宜于我科与相关科室平时即有多学科合作诊疗的基础。

肺癌根治术后早期并发急性高危PTE，一经确诊即有溶栓适应证，溶栓治疗的最大风险是手术部位的出血。为降低出血风险，我们认为应当选择在介入导管下行肺动脉内溶栓，同时辅以机械碎栓提高溶栓效率。溶栓过程中间断造影观察肺动脉灌注情况，一旦生命体征改善、主要肺血管血流恢复、肺动脉压力下降，应及时停用溶栓药并桥接肝素抗凝治疗。手术部位出血的监测应当由手术医生完成，如此前的肺叶切除手术中存在肺血管非机械性闭合的残端或破损，应当在溶栓前提出，并在介入溶栓过程中观察相应部位是否有活动性出血。初步治疗结束后需按计划给予足疗程的华法林抗凝治疗，对于存在DVT高危因素的患者应建议终生抗凝治疗。

PTE作为胸部外科围手术期常见且棘手的难题，应当被所有临床医生重视，我们建议有条件的医院应该制定成文的治疗方案，并安排多学科预演。对于急性高危PTE患者的诊疗原则、具体措施应该参照急性心梗的治疗建立绿色通道。
